# Distribution of petroleum degrading genes and factor analysis of petroleum contaminated soil from the Dagang Oilfield, China

**DOI:** 10.1038/srep11068

**Published:** 2015-06-18

**Authors:** Qinglong Liu, Jingchun Tang, Zhihui Bai, Markus Hecker, John P. Giesy

**Affiliations:** 1 Key Laboratory of Pollution Processes and Environmental Criteria (Ministry of Education), Tianjin Engineering Center of Pollution Diagnosis and Environmental Restoration, College of Environmental Science and Engineering, Nankai University, Tianjin 300071, China; 2 Research Center for Eco-Environmental Sciences, Chinese Academy of Sciences, Beijing 100085, China; 3 School of Environment and sustainability, University of Saskatchewan, Saskatoon, Saskatchewan, Canada; 4 Toxicology Centre, University of Saskatchewan, Saskatoon, Saskatchewan, Canada; 5 Department of Veterinary Biomedical Sciences, University of Saskatchewan, Saskatoon, Saskatchewan, Canada; 6 School of Biological Sciences, University of Hong Kong, Hong Kong, SAR, China; 7 State Key Laboratory of Pollution Control and Resource Reuse, School of the Environment, Nanjing University, Nanjing, People’s Republic of China; 8 Department of Biology, Hong Kong Baptist University, Hong Kong, SAR, China

## Abstract

Genes that encode for enzymes that can degrade petroleum hydrocarbons (PHs) are critical for the ability of microorganisms to bioremediate soils contaminated with PHs. Distributions of two petroleum-degrading genes *AlkB* and *Nah* in soils collected from three zones of the Dagang Oilfield, Tianjin, China were investigated. Numbers of copies of *AlkB* ranged between 9.1 × 10^5^ and 1.9 × 10^7^ copies/g dry mass (dm) soil, and were positively correlated with total concentrations of PHs (TPH) (R^2^ = 0.573, *p* = 0.032) and alkanes (C33 ~ C40) (R^2^ = 0.914, *p* < 0.01). The *Nah* gene was distributed relatively evenly among sampling zones, ranging between 1.9 × 10^7^ and 1.1 × 10^8^ copies/g dm soil, and was negatively correlated with concentrations of total aromatic hydrocarbons (TAH) (R^2^ = −0.567, *p* = 0.035) and ∑16 PAHs (R^2^ = −0.599, *p* = 0.023). Results of a factor analysis showed that individual samples of soils were not ordinated as a function of the zones.

Contamination of aquatic and terrestrial environments with petroleum hydrocarbons (PHs) represents a serious problem worldwide^1^. A total mass of between 8 × 10^4^ and 1 × 10^7^ tons of PHs per year have been estimated to be released into ecosystems globally^2^. Contamination of the environment with PHs is not uniform with locations where PHs are extracted, refined, or shipped typically being at greater risk of contamination. In China, there are more than 10 high-production oilfields, of which the Dagang Oilfield has 1.87 × 10^4^ square kilometers of exploration area with an annual production of 4.3 million tons of crude oil and 360 million cubic meters of gas, representing the major base for production, refining and shipping center in the Bohai Bay Rim area.

Alkanes and aromatic hydrocarbons (AHs) are the primary pollutants of concern associated with crude oil, and account for approximately 80% of the total petroleum hydrocarbons (TPHs) in crude oils^3^. Alkanes with carbon chain lengths ranging from C8 ~ C40 can cause hardening and limit wetting of soils, and can result in toxicity to plants and/or soil invertebrates, as well as pose risks to humans and wildlife through direct contact with these soils or soil organisms^4^. Another constituent of concern associated with crude oils are polycyclic aromatic hydrocarbons (PAHs), which have been shown to be of significant toxicological concern at environmental concentrations of more than 1000 μg kg^−1^, dm in soils^5^. PAHs can cause a range of toxic effects, including mutagenicity and carcinogenicity^6^,^7^. PAHs adhere to colloids of organic matter in soils, which can result in a decrease in bioavailability to PAHs-degrading microorganisms and an increase in recalcitrance of these compounds^8^.

Microbes that degrade petroleum hydrocarbons are widely distributed in the environment and constitute dynamic changes in structures of communities according to their divergent abilities biodegradate oils. Many indigenous oil-degrading microorganisms such as *Pseudomonas* GPo1 (C5–C12, n-alkanes)^9^, *Acinetobacter* sp. strain DSM17874 (C10–C40, n-alkanes)^10^, *Rhodococcus* sp. strain Q15 (C12–C32, n-alkanes)^11^, *Pseudomonas* putida G7 (naphthalene)^12^, *Burkholderia* sp. strain JS15 (BTEX)^13^ and *Pseudomonas* aeruginosa JI104 (toluene)^14^ and corresponding metabolic substrates have been characterized in detail. In addition, technologies based on abilities of certain microorganisms to enzymatically degrade PHs have been favored for remediation of soils because they are minimally invasive, require little disturbance of soils, are cost-effective and result in minimal secondary contamination^15^. Genes encoding for a variety of metabolizing enzyme systems that can degrade PHs play critical roles in aerobic mineralization activity, and may represent promising alternative approaches for the characterization of bioremediation capacities of microbes.

Alkane monooxygenase gene *AlkB* and naphthalene dioxygenase gene *Nah* are the predominant genes corresponding to first-step hydroxylases involved in metabolism of alkanes and AHs, respectively^16^,^17^. Previous studies have reported that indigenous microorganisms expressing alkane catabolic genes were able to degrade oil in contaminated soils or sediments^18^,^19^, even under extreme environmental conditions in the Arctic or Antarctic^20^,^21^. Alternatively, naphthalene dioxygenase can add both atoms of molecular oxygen to the aromatic ring as a first step in aerobic degradation, and subsequently transfer the corresponding dihydric alcohol to catechol^17^,^22^. Genes involved in degrading PHs have been successfully used as biomarkers for characterization of the magnitude of contamination of soils by PHs^18^,^23^. Abundance of *AlkB* and PAH-ring-hydroxylating dioxygenases genes have been shown to be positively correlated to most probable number (MPN) counts of microorganisms that can degrade PHs, and to degradation of hexadecane and naphthalene^24^. Understanding relationships between abundance/expression of genes and biodegradation of PHs is important for predicting potential effects of contamination ecosystems with oil and the metabolic remediation capability of aerobic soil microorganisms.

This study was designed to characterize major classes of constituents in crude oil including total petroleum hydrocarbons (TPHs), saturated hydrocarbons (SHs), aromatic hydrocarbons (AHs) and concentrations of other components of TPHs in soils in three zones of the Dagang oilfield. To evaluate the presence and metabolic potential of oil degrading microorganisms, Q-PCR was used to quantify abundances of *AlkB* and *Nah*. Microbial communities were also characterized by use of PCR and Denaturing Gradient Gel Electrophoresis (DGGE). Finally, multivariate statistical techniques were applied to characterize relationships between numbers of copies of oil degrading genes, soil parameters as well as concentrations of PHs.

## Results

### Petroleum contamination and other physico-chemical properties

Soils collected from the Dagang Oilfield were variously contaminated with PHs (Table 1). The range in concentrations of TPHs, as measured gravimetrically in the three zones were: oil-producing zone, (2.0 ± 0.5) × 10^4^ ~ (3.7 ± 0.7) × 10^4^ mg kg^−1^ dm.; residential zone, (1.3 ± 0.3) × 10^4^ ~ (3.4 ± 0.5) × 10^4^ mg kg^−1^ dm.; oil-refinery and transportation zone, (2.0 ± 0.5) × 10^4^ ~ (2.3 ± 0.4) × 10^4^ mg kg^−1^ dm. Concentrations of TPH at S1 and S2 less than10 meters away from the oil well, as well as S8 near oil storage tanks in the residential zone exceeded 3.0 × 10^4^ mg kg^−1^ dm, which were significantly greater than those at all other sites (*p* < 0.05). Concentrations of TPHs in soils near the oil tank and other petroleum transportation routes were greater than those in soils along the canal and in the residential area. Due to the run-off affect, soils collected next to the Banqiao canal at S6 contained the lesser concentration of TPHs with (1.3 ± 0.3) × 10^4^ mg kg^−1^ dm.

Concentrations of TPHs in soils collected from oil-refinery and transportation zones, where there were fixed transport routes and oil-refinery workshops, were more homogeneous compared to the other zones, with concentrations ranging from (2.0 ± 0.5) × 10^4^ to (2.3 ± 0.2) × 10^4^ mg kg^−1^ dm. The variance of TPHs among the three sampling zones was examined by LSD-t method, and found that there was no significant difference between the oil producing zone and other zones.

SHs and AHs were the primary constituents of PHs (Table S1). Proportions of total hydrocarbons (THCs) contributed by SHs in the oil-producing, residential and oil-refinery and transportation zones were 23.3%, 20.5%, 34.1%, respectively, and those derived from AHs were 13.3%, 8.12% and 13.2%, respectively. The greatest concentration of SHs (1.1 ± 0.2) × 10^4^ mg kg^−1^ dm was observed at S1, and the least concentration of (2.6 ± 0.8) × 10^3^ mg kg^−1^ dm was observed at S4. S11 had the greatest concentrations of TAHs (5.4 ± 0.2) × 10^3^ mg kg^−1^ dm, and the least concentration of (1.1 ± 0.5) × 10^3^ mg kg^−1^ dm was observed at S8.

The 35 alkanes (C8 ~ C40) consisted predominantly of carbon chain lengths of C8 ~ 32, with C8 ~ C19 and C20 ~ C32 alkanes, which accounted for 47.0% and 50.4%, respectively. Concentrations of alkanes (C8 ~ C40) in the residential zone, ranging from 2.8 ± 0.2 × 10^2^ to 7.2 ± 0.7 × 10^3^ μg kg^−1^ dm, showed a significantly lesser range compared those in the oil producing zone, which ranged from 1.9 ± 0.1 × 10^4^ to 6.6 ± 0.2 × 10^3^ μg kg^−1^ dm, and oil-refinery and transportation zones, where they ranged from (1.8 ± 0.2) × 10^4^ to (1.3 ± 0.1) × 10^4^ μg kg^−1^ dm) (*p* < 0.01). However, there was no significant difference in concentrations of ∑16 PAHs between the oil-producing zone, where they ranged from (1.7 ± 0.1) × 10^2^ to (1.3 ± 0.05) × 10^3^ μg kg^−1^ dm, residential zone, where they ranged from (1.9 ± 0.2) × 10^2^ to (4.6 ± 0.4) × 10^2^ μg kg^−1^ dm) and oil-refinery and transportation zone, ranging from (1.8 ± 0.2) × 10^2^ to (2.8 ± 0.3) × 10^3^ μg kg^−1^ dm. PAHs were composed mainly of naphthalene, phenanthrene and pyrene. These three PAHs constituted approximately 55.7% of ∑16 PAHs in analyzed soils.

Values of pH of soils of the Dagang oilfield ranged from 8.38 to 8.63, and were homogeneous among zones. Salt contents of soils from different zones was more variable with concentrations ranging from 1.42 to 53.83 g kg^−1^ dm. There were significant (p < 0.05) differences in salinity between those of the residential zone and other zones, with a large variability of salinity in the residential zone where concentrations ranged from 3.32 to 58.83 g kg^−1^ dm. Compared with the salt content of other major Chinese oilfields: Shengli (9.8 g kg^−1^ dm), Changqing (4.3 g kg^−1^ dm), Daqing (7.6 g kg^−1^ dm), Yumen (4.4 g kg^−1^ dm), Jianghan (5.3 g kg^−1^ dm)^25^, the Dagang oilfield had markedly greater salinities with an average of 16.47 g kg^−1^ dm in this study.

### Distribution of degradation genes

**Figure 1:**
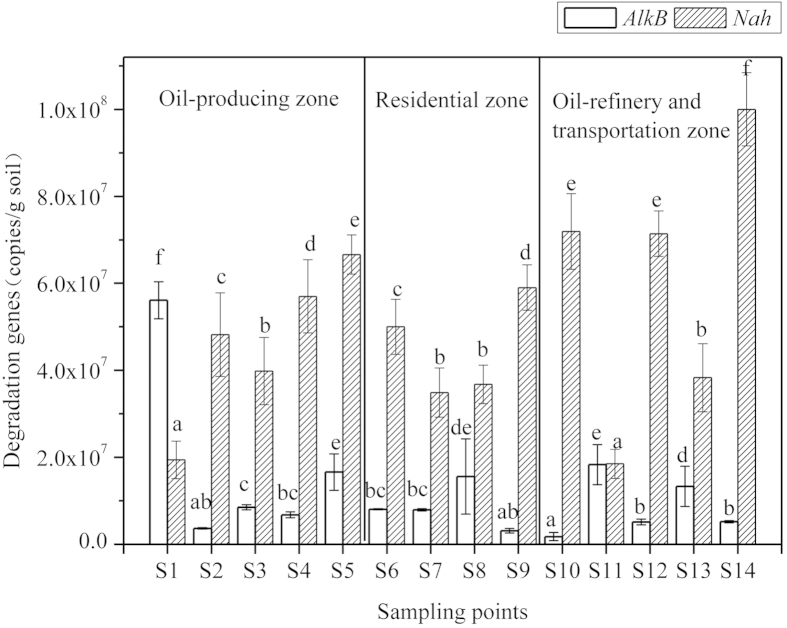
Distribution of *AlkB* and Nah degradation genes in all the Dagang Oilfield soil samples. Error bars represent standard deviations of efficiencies for PCR. Letters on the columns indicate a significant difference among sites at p < 0.05 according to Duncan’s multiple range tests of One-Way ANOVA.

Numbers of copies of genes *AlkB* and *Nah*, coding for PH-degrading enzymes, that were detected by qPCR in soils of the Dagang Oilfield differed significantly among sampling sites (Fig. 1), and the relative abundance of *AlkB* and *Nah* genes were normalized to total 16S rDNA (Table S2). The standard curve for quantification of numbers of copies of PH-degrading genes (*AlkB* and *Nah*) and the 16S rDNA is presented in the supplementary materials (Figure S1). Abundances of *AlkB* exhibited large variations in different oil contaminated areas, ranging from (1.8 ± 0.9) × 10^6^ to (5.6 ± 0.4) × 10^7^ copies/g dm soil. Numbers of copies of *AlkB* in soils collected from S1, S5, S8, S11 and S13 were significantly (p < 0.05) greater than those at other sampling sites. The residential zone, which had a lesser concentration of alkanes, exhibited a lesser relative abundance of *AlkB*. Mean relative abundances of *AlkB* were as follows: oil-producing zones (3.0 ± 0.4) × 10^−3^ > oil-refinery and transportation zones (2.3 ± 0.3) × 10^−3^ > residential zones (4.0 ± 0.3) × 10^−4^.

Numbers of copies of *Nah* ranged from (1.9 ± 0.5) × 10^7^ to (1.0 ± 0.08) × 10^8^ copies/g dm soil, and abundance of *Nah* at S4, S5, S10, S12, S14 significantly (*p* < 0.05) greater than other sites. Distribution of *Nah* was relatively even across soils of oil-producing, residential, and oil-refinery and transportation zones ((4.6 ± 0.7) × 10^7^, (4.5 ± 0.5) × 10^7^, (6.2 ± 0.6) × 10^7^ copies/g dm soil, respectively). However, the relative abundance of Nah fluctuated among S9 with the least value of (2.9 ± 0.4) × 10^−4^ and S14 with the greatest value of (8.1 ± 0.5) × 10^−2^.

### DGGE analysis

**Figure 2:**
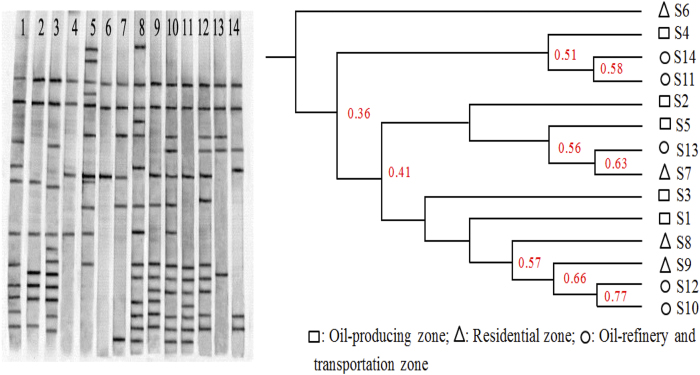
DGGE analysis result of different soil samples in Dagang oil field. (**a**) Community profiles based on DGGE analysis of the V3 region of the 16S rDNA amplified by PCR of DNA extracts from soil samples of S1 to S14 (lanes 1–14). (**b**) Dendrogram analysis (UPGMA) of DGGE banding patterns from different soil samples.

Diversity of microbial communities in soils, based on DGGE, was estimated by the number of amplified 16S rDNA bands (Fig. 2a), in which each band was assumed to represent a single operational taxonomic unit (OTU)^26^. Samples S1, S2, S3, S8, S9 and S10 displayed a greater number of bands, whereas samples S4, S6, S7 and S13 produced fewer distinct bands. Partitioning of features of microbial community structure can be seen in the dendrogram that was created by illustrating the similarity of microbial communities in soils (Fig. 2b). Microbial communities could be grouped into three major phylogenetic clusters. S10 and S12, which both were located in the oil refinery and transportation zones, revealed similar community structure with the highest similarity (85.9%), whereas S6 and S10 displayed the divergent communities with only 17.2% similarity. Moreover, the soils in the same zones with different concentrations of PHs and salinity were grouped into different clusters.

Values of the Shannon-Wiener Index of 14 soils from the Dagang Oilfield were between 1.5 and 3, and Uniformity Indexes were almost equivalent in each soil (Table S3). In general, greater Shannon-Wiener Indexes of petroleum contaminated soils at locations S1, S2, S5, S8 and S10 were more similar to soils S3 and S9, which had lesser salinities and differ greatly from soils S6, S7 and S14, which had greater salinities.

### Relationship between degradation genes and other parameters

**Figure 3:**
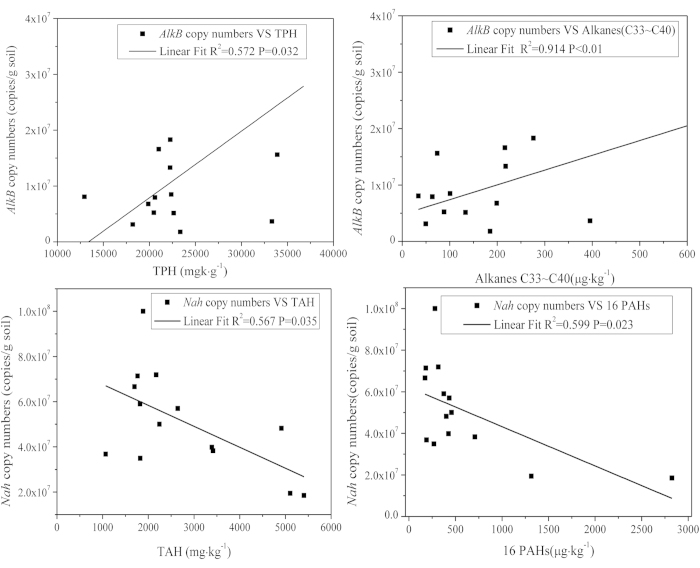
Correlations between numbers of copies of genes involved in degradation with the responding petroleum components.

Numbers of copies of *AlkB* were positively correlated with various petroleum components, including TPHs (R^2^ = 0.573, *p* = 0.032) and long-chain alkanes (C33 ~ C40) (R^2^ = 0.914, *p* < 0.01) (Fig. 3). Soils collected from the oil-producing zone, which contained greater concentrations of alkanes exhibited the greatest numbers of copies of *AlkB.* Numbers of copies of *Nah* were negatively correlated with TAH (R^2^ = −0.567, *p* = 0.035) and ∑16 PAHs (R^2^ = −0.599, *p* = 0.023) (Fig. 3).

### Factor analysis

**Figure 4:**
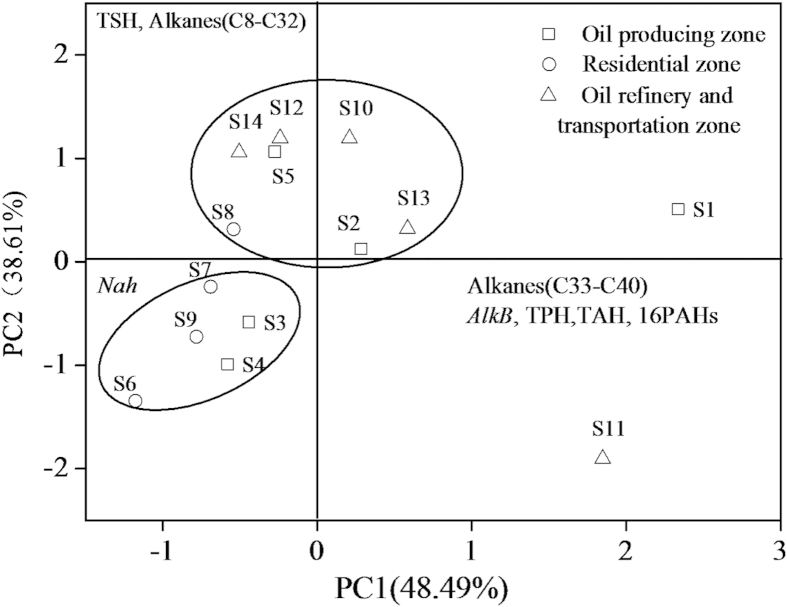
Factor analysis of degradation genes and the petroleum components. PC1 and PC2: first and second principal components in the analysis

When the eleven measures of contamination of soils by PHs and two classes of genes involved in degradation of PHs were analyzed by factor analysis, the first two principal components explained 73.7% of the total variance (Fig. 4). Sites S1 and S11 were ordinated differently from all other locations, which indicated different sources of PHs. S1 was the most contaminated site, and had the greatest abundance of *AlkB*. S6, S7, S9 in soils of the residential zone as well as S3, S4 grouped together in the lower left quadrants of the graph, an area that was characterized by greater concentrations of lesser molecular weight PHs and a general accumulation of microorganisms containing the *Nah* gene. Locations S2, S5, S8, S10, S12, S13 and S14 were ordinated together in the upper portion of the diagram, which indicated that these samples were predominated by AHs and alkanes (C8 ~ C32). Copies of *AlkB* had larger factor scores compared with abundance of *Nah* in these areas. Even though some sites, such as S2, S3, S4 and S5 were relatively close together, they were ordinated differently except samples from the residential zone, which were clustered together. Factor analysis showed that different sites were not plotted according to the located zones.

## Discussion

The correlation between abundance of oil-degrading genes and oil pollution in different zones represented a useful method for describing factors involved in bioremediation soils contaminated with oil. Numbers of copies of the *AlkB* and *Nah* genes in soils differed among the three zones. Soils in the residential zone with lesser concentrations of alkanes and relative abundance of *AlkB* might be explained by lesser oil emission and leakage than other sampling sites. Dagang Oilfield soils exhibited abundances of *AlkB* similar to those in soils from other oil exploring areas, and greater than those in sediment contaminated with crude oil that were investigated previously. The number of copies of the *AlkB* gene varied from 2.56 × 10^6^ to 9.37 × 10^7^ copies per gram dry soil in Daqing Oilfield soils, and 7.48 × 10^5^ to 6.63 × 10^7^ copies per gram dry soil in Karamay Oilfield soils^27^. The number of copies of the *AlkB* gene ranged from 1.1 × 10^5^ to 2.9 × 10^5 ^copies g^−1^ in Timor Sea sediment contaminated with oil^28^.

Compared with numbers of copies of *AlkB*, numbers of copies of *Nah* were approximately 10-fold greater in soils of all three zones. Multimeric, naphthalene dioxygenase was the major enzyme involved in aerobic metabolism of naphthalene that can also mineralize phenanthrene, BTEX and other PAHs through ring-opening, and terminal oxidation^29^. The *Nah* degradation gene is generally found in soils and sediments contaminated with PHs. However, the terminal monooxygenase encoded by the *AlkB* gene has greater specificity in degradation of constituents of PHs than the enzyme encoded by the *Nah* gene, and degradation targets are primarily short and medium-chain alkanes (C6 ~ C15)^16^,^23^,^30^,^31^,^32^. Although the alkane monooxygenase enzyme can also mineralize longer-chain alkanes (C30 ~ C40), appropriate sets of primers were designed for specific PHs in particular soils^28^,^33^. In conclusion, naphthalene dioxygenases with a wider range of target substrates compared to alkane monooxygenase are likely to be more common in oil contaminated soils, and thus, are hypothesized to have resulted in greater numbers of copies of *Nah* reported here.

Differences in numbers of DGGE bands suggest that variations in bacterial communities might be affected synthetically by concentrations of petroleum and salinity. Communities of microbes in S1, S2, S8, S9 and S10 with greater species diversity were selectively enriched by higher concentration of petroleum. Soils from S6, S7 and S14, which had lesser species diversities were affected by greater salinity, whereas, S3 and S9 which had lesser salinities, exhibited richer biodiversity, even though both of these sites had lesser concentrations of PHs. A previous study identified only a few halophilic microbes, including *Halomonas* and *Dietzia*, by use of polymorphism community fingerprinting PCR in saline soils with 20% NaCl near Comodoro Rivadavia in Patagonia that were contaminated with diesel fuel^30^. Other studies have found inhibition of growth of microbes and diversities of genes involved in degradation of PHs and a decrease in oil degradation rates at sites with a greater salt content (>5% NaCl)^31^,^34^. Thus, greater salinity and pH are likely limiting factors for bioremediation PHs in soils of the Dagang. The diversity indexes observed were consistent with the conclusion that the presence of greater concentrations of TPHs enriched the microbial community, while salinity tended to inhibit the microbial communities of soils.

Numbers of copies of genes, quantified by Q-PCR, that are involved in degradation of PH, can be used as the biomarker to reflect the bioremediation potential of microbes in oil contaminated soils. The statistically significant, positive correlation between the number of copies of the *AlkB* gene and concentrations of alkanes clearly illustrated that oil-degrading microorganisms containing *AlkB* gene were a good indicator of long-term exposure to alkanes in contaminated soils. This conclusion is consistent with that of Pérez-de-Mora *et al.* who found a positive correlation between alkane contents and the abundance of *AlkB* in soils at four forest sites co-contaminated with mineral oil hydrocarbons and metals^35^. Genes involved in degrading longer-chain n-alkane were also found in soils contaminated with PHs^33^,^36^.

Abundances of *Nah* genes was between 3 × 10^1^ and 9 × 10^4^ copies/g dm soil at sites in the vicinity of Trollberget, Etna and Sköldvik, southern Finland and were positively correlated with the rate of aerobic mineralization of ^14^C-naphthalene^37^. S1 and S11, which had the greatest abundances of 16 PAHs, also had the least number of copies of *Nah*. This result indicated that greater concentrations of PAHs resulted in lesser abundance of microorganisms capable of degrading PHs. Soils with lesser concentrations of naphthalene (10 μg g^−1^) exhibited greater expression of naphthalene dioxygenase during vermicompost remediation for 30 days, while greater naphthalene concentrations of 100 μg g^−1^ led to lesser expression of naphthalene dioxygenase^38^. It is hypothesized that the greater concentrations of PAHs observed in soils at sites S1 and S11 were toxic to certain microbes that express the *Nah* gene, and thus, resulted in a lesser abundance of this gene.

In this study, correlations between oil degradation genes (*AlkB* and *Nah*) and different oil pollution in oil producing, residential, and oil-refinery and transportation zones was studied. Concentration of oil and salinity both affected expression of genes involved in degradation of oil as well as biodiversity of indigenous microorganisms. Dynamic quantification of oil degradation genes can be used as biomarker to estimate the bioremediation capacity of indigenous microbes in oil contaminated soils. In addition, metabolic enzymes encoded by responding oil degradation genes played crucial roles in the oil degradation process. Therefore, further investigation of the enzyme activity is of great significance.

## Materials and Methods

### Hydrocarbon-contaminated soils and sampling

**Figure 5:**
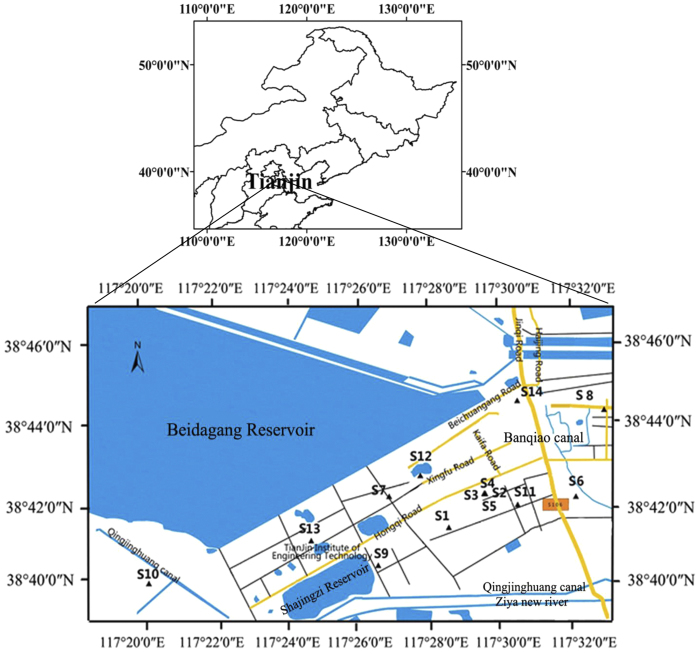
Sampling area and location of sampling points. The figure map was generated by using software ArcGIS 10 (Environmental Systems Research Institute, Inc. Redlands, US). The sampling sites were located by using Global Positioning System (GPS).

Fourteen soils contaminated with PHs were sampled from three different zones in the Dagang Oilfield area, which is situated in the southeast of Tianjin city, Northern China (lat.38°39′47.43″ ~ 38°44′42.37″N, long.117°20′1.47″ ~ 117°32′41.30″E): oil-producing, S1–S5 (Distance from the well: S1, S2 < 10 m; 20 < S3–S5 < 50 m); residential, S6–S9; and oil-refinery and transportation zones, S10–S14 (Table 2; Fig. 5). Soils were collected to a depth of ~10–20 cm. Four samples of soil were collected at each site using cross sampling methods by use of sterile spatulas. These four sub-samples were combined and then thoroughly homogenized to obtain a uniform composite for each site with total weight of 4 kg. Samples were transported to the laboratory on ice and stored at −20 °C for microbial analysis (DNA extraction and genetic characterization). Samples were air-dried for the determination of PHs and other physical and chemical properties such as pH and salt content.

### Measurement of salinity and pH

Salinity was determined by use of a gravimetric method. In brief, a suspension of soil (1:5 soil: deionized water, w:w) was heated in water bath at 100 °C to dryness, and 10% H_2_O_2_ was added as oxidant subsequently for three times. The residual was then weighted and salinity was calculated. The pH of the soil suspension was measured by a pH meter (Sartorius, Germany).

### Gravimetric quantification of total petroleum hydrocarbon (TPH)

Five-gram aliquots of soils were Soxhlet-extracted for 18 h with 125 ml dichloromethane at 54 °C^39^,^40^. Extracts were concentrated to dryness by use of a rotary evaporator, and concentrations of TPHs were determined gravimetrically^41^. The increment in mass of round-bottom flasks after evaporation of the extracts was defined as the final TPHs concentration. All extracts were analyzed in triplicate.

### Gravimetric identification and quantification of components of PHs

Methods for purification and separation of constituents of PHs were modified from those previously published^42^,^43^. In brief, concentrated extracts were eluted from a glass column (dimensions: 20 mm × 400 mm) containing pre-rinsed activated silica gel and neutral aluminum (12 g:6 g, soaked with hexane) using the following sequence of solvents: 20 mL of hexane, 70 mL of hexane and dichloromethane (1:1), 50 mL of methanol to separate SHs, AHs and polar components. After purification and separation, the three solvent fractions were reduced to dryness and concentrations of PHs were determined by gravimetric measurement of the extracted residues as described above.

### Quantification of PAHs and saturated hydrocarbons by GC/MS

Concentrations of PAHs and SHs were determined by use of a 6850 Agilent HP gas chromatograph connected to a 5975 Agilent HP mass spectrometer (Agilent, CA, USA). Components of PHs were separated by a Thermo Trace GC Ultra system equipped with a Thermo DB-5MS capillary column coated with 5% diphenyl and 95% dimethyl polysiloxane stationary phase (30 m × 0.25 mm, i.d. 0.25 μm film thickness; Thermo Scientific, Runcorn, UK), operating with helium (99.99% purity) as the carrier gas at a constant flow of 1.0 ml min^−1^. 1 μl aliquots of each were injected at 280 °C in pulsed, splitless mode (1 min, then split ratio 1:50 to the end of analysis). The GC oven temperature was held at 60 °C for 3 min, and then temperature was increased by 15 °C/min from 60 °C to 180 °C, followed by 6 °C/min from 180 °C to 300 °C, and then temperature was held at 300 °C for 10 min. The mass spectrometer was operated with the ion source at 220 °C with an ionization energy of 70 eV. Five different concentrations of mixtures of 16 target PAHs and 33 target alkanes (C8 ~ C40) were used as external standards for determination of components of extracts. PAHs and SHs were quantified in single ion monitoring (SIM) mode, with the molecular ion of each PAH and SH component corresponding to the elution retention time of the external standard.

### Quantification of petroleum degrading genes and 16S rDNA

Real-time q-PCR based on fluorescent dye SYBR green I was used to quantify two PH degradation genes (*AlkB* and *Nah*) and 16S rDNA. Degenerated primer sets, designed for *AlkB* amplication in 17 microbes and *Nah* amplication in 33 PAHs degradation bacterial species were used according to previously studies^44^,^45^. Primer sets for 16S rDNA were adapted from Suzuki^46^. The sequence, amplicon size and annealing temperature conditions of the PCR primers are given (Table S4).

DNA was extracted from soils by use of a ZR Soil Microbe DNA MiniPrep™ (Zymo Research, USA) following the protocol provided by the manufacturer. Conventional PCR was used to verify and recover the target genes. Each PCR reaction mixture contained 10× Easy Taq buffer, 0.2 mM total concentrations of dNTPs mixture, 5 mU Easy Taq DNA polymerase, 0.4 μmol of both forward and reverse primers, and 2 μL DNA per plate. Conditions for conventional PCR were set according to previously published methods^47^: initial denaturation at 94 °C for 5 min; 35 cycles of denaturing at 94 °C for 20 s, annealing temperature of target genes were set as Table S1, for 2 min; and final extension at 72 °C for 7 min. PCR products were examined by 1.5% agarose gel electrophoresis.

Target genes were recovered from agarose gel by using gel extraction kit (Axygen, USA), and were combined with pEASY-T1 vectors (Trans Gen Biotech, China) and transformed into Escherichia coli JM109 (Takara, Japan). Plasmids carrying target genes were extracted with Plasmid kit (Omega, USA) and quantified by use of a nucleic acid analyzer (Bibby Scientific Limited, United Kingdom) to construct standard curves. Because lengths of the vector and target gene inserts were known, gene copy numbers were calculated directly from extracted plasmid DNA concentration. For every set of primers, standard curves were obtained from ten-fold serial dilutions of template DNA prepared from plasmids containing target genes (Figure S1; Supplemental Materials).

Quantification of 16S rDNA, *AlkB*, and *Nah* degradation genes by qPCR were carried out on a BioRad CFX96 (Hercules, USA) with a C1000 thermal cycler iCycler by using Quantitect^®^ SYBR green PCR kits (Trans Gen Biotech, China) following the manufacturer’s directions. Reaction mixtures contained 12.5 μL 2 × trans start^TM^ top green qPCR super mix, 0.5 μL passive reference dye, and 1 μL of recombinant plasmids DNA and soil DNA as template for the construction of standard curve and quantification of target genes, respectively. Concentrations of primers were optimized to 0.2 μmol for both the forward and reverse primer. Cycling conditions for real-time qPCR were as follows: hold for 30 s at 94 °C followed by 40 cycles of denaturing at 94 °C for 5 s, annealing temperature of primers target genes were set as Table S4, for 15 s, extension and first plate read at 72 °C for 10 s; hold at 55 °C for 30 s; a second plate read at 55 °C hold for 5 s followed by a melt curve from 55 °C to 95 °C (increment = 0.5 °C/10 s).

Every template plasmid DNA was run in triplicate and each experiment was repeated at twice in order to generate a reproducible dataset and avoid false detections in environmental samples. Melt curve analyses were used to detect the formation of primer dimmers and other amplification of nonspecific sequences. Data were analyzed with CF Manager Software (version 2.1, Bio-Rad, US). The limit of detection for quantification of each gene was determined by comparing the linear relationship between the base-10 logarithm of diluted concentrations of the plasmid DNA and the fluorescence signal.

### Analysis of microbial community by PCR-DGGE

PCR- DGGE technology was adopted to assess the microbial community of soil samples targeting the V3 region of bacterial 16S rDNA. The primer set targeting the V3 region of bacterial 16S rDNA consisted of GC-338f (5′-GCclamp- CACGGGGGGACTCCTACGGGAGGCAGCAG-3′) (GC clamp = CGCCCGCCGCGCGCGGCGGGCGGGGCGGGGGCACGGGGGG) and 518r (5′-ATTACCGCGGCTGCTGG-3′). PCR amplicons were loaded with loading dye into 8% polyacrylamide gel (37.5:1 acrylamide: bisacrylamide) with a denaturing gradient from 15% to 45% (100% denaturant consisted of 7 M urea and 40% formamide, v/v). The DGGE analysis was carried out on a Universal Mutation Detection System D-code (BioRad, CA, USA) at140 V and 60 °C for 3 h. Gels were then stained with ethidium bromide, visualized with an UV transilluminator (ATTO Corporation, Japan). Dendrogram and intensities analysis of DGGE banding patterns was performed using Quantity One 4.6 (Bio-Rad Laboratories, CA, USA). Calculation of the pair-wise similarities was based on the Dice correlation coefficient. Dendrograms were created using the algorithm of un-weighted pair-group method with the arithmetic averages (UPGMA)^48^.

### Statistical analysis

All mathematical and statistical computations were conducted using SPSS 16.0 (IBM, New York, USA). Comparison of concentrations of PHs among sites was accomplished by use of One-Way ANOVA. Differences among zones were analyzed by use of the nonparametric, Kruskal-Wallis test. Normality was confirmed by the Kolmogorov-Smirnov test and homogeneity of variance was confirmed by use of Levine’s test. In order to further investigate distributions of degradation genes and the correlation with the petroleum pollution status of Dagang Oilfield soils, 13 factors were measured to characterize soils. Inter-relationships were analyzed by factor analysis (FA) on the varimax-rotated factors, and factor loadings were calculated by use of eigenvalues greater than 1.0^49^.

## Additional Information

**How to cite this article**: Liu, Q. *et al.* Distribution of petroleum degrading genes and factor analysis of petroleum contaminated soil from the Dagang Oilfield, China. *Sci. Rep.* **5**, 11068; doi: 10.1038/srep11068 (2015).

## Supplementary Material

Supplementary Information

## Figures and Tables

**Table 1:** Characteristics of petroleum hydrocarbons (PHs) in soils of the Dagang oilfield.

**Samples**		**TPH (mg kg^−1^ dm)**	**TSH (mg kg^−1^ dm)**	**TAH (mg kg^−1^ dm)**	**Alkanes (C8 ~ C40)(μg kg^−1^ dm)**	**16 PAHs (μg kg^−1^ dm)**	**naphthalene (μg kg^−1^ dm)**	**phenanthrene(μg kg^−1^ dm)**	**pyrene(μg kg^−1^ dm)**	**Alkanes (C8 ~ C19) (μg kg^−1^ dm)**	**Alkanes (C20 ~ C32) (μg kg^−1^ dm)**	**Alkanes (C33 ~ C40) (μg kg^−1^ dm)**	**pH**	**salinity(g kg^−1^ dm)**
Oil-producing zone	S1	(3.7 ± 0.7) × 10^4^	(1.1 ± 0.2) × 10^4^	(5.1 ± 0.8) × 10^3^	(1.9 ± 0.1) × 10^4^	(1.3 ± 0.05) × 10^3^	(1.2 ± 0.2) × 10^2^	7.7 × 10^1^	2.5 × 10^2^	7.7 × 10^3^	9.8 × 10^3^	1.9 × 10^3^	8.55	16.56
	S2	(3.3 ± 0.7) × 10^4^	(6.5 ± 0.3) × 10^3^	(4.9 ± 0.2) × 10^3^	(1.1 ± 0.1) × 10^4^	(4.0 ± 0.3) × 10^2^	(7.9 ± 1.2) × 10^1^	7.4 × 10^1^	8.2 × 10^1^	4.1 × 10^3^	7.0 × 10^3^	4.0 × 10^2^	8.54	7.97
	S3	(2.2 ± 0.6) × 10^4^	(3.5 ± 0.4) × 10^3^	(3.4 ± 0.5) × 10^3^	(6.6 ± 0.2) × 10^3^	(4.3 ± 0.2) × 10^2^	(5.9 ± 0.9) × 10^1^	8.9 × 10^1^	6.9 × 10^1^	3.2 × 10^3^	3.2 × 10^3^	1.0 × 10^2^	8.53	7.10
	S4	(2.0 ± 0.5) × 10^4^	(2.6 ± 0.8) × 10^3^	(2.6 ± 0.4) × 10^3^	(1.2 ± 0.1) × 10^4^	(4.0 ± 0.7) × 10^2^	(1.2 ± 0.0) × 10^2^	8.3 × 10^1^	7.0 × 10^1^	8.1 × 10^3^	3.7 × 10^3^	2.0 × 10^2^	8.39	8.76
	S5	(2.1 ± 0.2) × 10^4^	(7.8 ± 0.8) × 10^3^	(1.7 ± 0.3) × 10^3^	(1.3 ± 0.1) × 10^4^	(1.7 ± 0.1) × 10^2^	(2.7 ± 0.1) × 10^1^	3.9 × 10^1^	5.1 × 10^1^	6.3 × 10^3^	6.9 × 10^3^	2.2 × 10^2^	8.63	11.78
Residential zone	S6	(1.3 ± 0.3) × 10^4^	(9.7 ± 0.9) × 10^2^	(2.2 ± 0.6) × 10^3^	(2.8 ± 0.2) × 10^2^	(4.6 ± 0.4) × 10^2^	(5.8 ± 0.6) × 10^1^	7.1 × 10^1^	1.0 × 10^2^	1.4 × 10^2^	1.1 × 10^2^	3.4 × 10^1^	8.51	53.83
	S7	(2.1 ± 0.5) × 10^4^	(5.4 ± 0.8) × 10^3^	(1.8 ± 0.4) × 10^3^	(5.9 ± 0.1) × 10^3^	(2.7 ± 0.3) × 10^2^	(4.7 ± 0.9) × 10^1^	7.4 × 10^1^	3.6 × 10^1^	3.6 × 10^3^	2.3 × 10^3^	6.3 × 10^1^	8.43	26.72
	S8	(3.4 ± 0.5) × 10^4^	(5.2 ± 0.4) × 10^3^	(1.1 ± 0.5) × 10^3^	(7.2 ± 0.7) × 10^3^	(1.9 ± 0.2) × 10^2^	(3.7 ± 0.2) × 10^1^	4.6 × 10^1^	2.9 × 10^1^	4.4 × 10^3^	2.7 × 10^3^	7.4 × 10^1^	8.56	33.23
	S9	(1.8 ± 0.2) × 10^4^	(6.0 ± 1.4) × 10^3^	(1.8 ± 0.3) × 10^3^	(2.9 ± 0.3) × 10^3^	(3.7 ± 0.1) × 10^2^	(1.3 ± 0.1) × 10^2^	6.6 × 10^1^	6.3 × 10^1^	2.0 × 10^3^	9.1 × 10^2^	5.0 × 10^1^	8.44	3.32
Oil-refinery and transportation zone	S10	(2.3 ± 0.4) × 10^4^	(9.0 ± 1.3) × 10^3^	(2.2 ± 0.5) × 10^3^	(1.8 ± 0.2) × 10^4^	(3.2 ± 0.3) × 10^2^	(8.3 ± 0.7) × 10^1^	7.6 × 10^1^	6.3 × 10^1^	9.5 × 10^3^	8.6 × 10^3^	1.9 × 10^2^	8.38	4.82
	S11	(2.2 ± 0.1) × 10^4^	(6.0 ± 0.8) × 10^3^	(5.4 ± 0.2) × 10^3^	(1.5 ± 0.4) × 10^4^	(2.8 ± 0.3) × 10^3^	(1.3 ± 0.1) × 10^2^	2.6 × 10^2^	4.4 × 10^2^	6.0 × 10^3^	8.4 × 10^3^	2.8 × 10^2^	8.41	14.37
	S12	(2.3 ± 0.2) × 10^4^	(7.6 ± 0.8) × 10^3^	(1.8 ± 0.2) × 10^3^	(1.5 ± 0.04) × 10^4^	(1.8 ± 0.2) × 10^2^	(3.7 ± 0.5) × 10^1^	3.9 × 10^1^	5.3 × 10^1^	7.6 × 10^3^	7.6 × 10^3^	1.3 × 10^2^	8.61	13.48
	S13	(2.2 ± 0.3) × 10^4^	(8.5 ± 0.8) × 10^3^	(3.4 ± 0.6) × 10^3^	(1.6 ± 0.1) × 10^4^	(7.1 ± 0.5) × 10^2^	(8.8 ± 1.1) × 10^1^	1.1 × 10^2^	1.1 × 10^2^	7.7 × 10^3^	8.0 × 10^3^	2.2 × 10^2^	8.4	1.42
	S14	(2.0 ± 0.5) × 10^4^	(6.6 ± 0.9) × 10^3^	(1.9 ± 0.9) × 10^3^	(1.3 ± 0.1) × 10^4^	(2.8 ± 0.7) × 10^2^	(4.0 ± 0.9) × 10^1^	5.4 × 10^1^	6.2 × 10^1^	7.1 × 10^3^	6.1 × 10^3^	8.8 × 10^1^	8.62	27.32

Mean ± standard deviation for triplicate experiments.

**Table 2:** Latitude and longitude of sampling sites in Dagang Oilfield.

**Zones**	**Sampling number**	**Sampling sites**	**Latitude**	**Longitude**
Oil-producing zone	S1	Chuangxin Road	38°41′17.92″	117°28′22.24″
	S2	Lesser zone	38°42′12.80″	117°29′21.14″
	S3	Middle zone	38°42′12.69″	117°29′21.52″
	S4	Higher zone	38°42′12.39″	117°29′22.39″
	S5	Test field zone	38°42′12.53″	117°29′23.10″
Residential zone	S6	Chuangxin Road-canal	38°42′8.06″	117°31′54.31″
	S7	Xingfu Road-pool	38°42′7.77″	117°26′42.61″
	S8	Chuangye Road-oil tank	38°44′28.13″	117°32′41.30″
	S9	Chuangxin Road	38°40′16.79″	117°26′24.37″
Oil-refinery and transportation zone	S10	Maxi Road	38°39′47.43″	117°20′1.47″
	S11	Chuangxin Road-living zones	38°41′54.77″	117°30′17.01″
	S12	Xingfu Road1	38°42′41.63″	117°27′34.44″
	S13	Xingfu Road2	38°40′56.46″	117°24′32.47″
	S14	Yard 2 bridge	38°44′42.37″	117°30′16.16″
