# Supplementary material for: Distribution of petroleum degrading genes and factor analysis of petroleum contaminated soil from the Dagang Oilfield, China
Source: Sci Rep. 2015 Jun 18;5:11068. doi: 10.1038/srep11068 (PMC4478889; doi:10.1038/srep11068)
Supplement: Supplementary Information [file srep11068-s1.doc]

**Distribution of petroleum degrading genes and factor analysis of petroleum contaminated soil from the Dagang Oilfield, China**

Qinglong Liu^1^, Jingchun Tang^1*^, Zhihui Bai^2^, Markus Hecker^3,4^ and John P. Giesy^4,5,6,7,8,9,10^

^1^ Key Laboratory of Pollution Processes and Environmental Criteria (Ministry of Education), Tianjin Key Laboratory of Environmental Remediation and Pollution Control, College of Environmental Science and Engineering, Nankai University, Tianjin 300071, China

^2^ Research Center for Eco-Environmental Sciences, Chinese Academy of Sciences, Beijing 100085, China

^3^ School of Environment and sustainability, University of Saskatchewan, Saskatoon, Saskatchewan, Canada.

^4^ Toxicology Centre, University of Saskatchewan, Saskatoon, Saskatchewan, Canada.

^5^ Department of Veterinary Biomedical Sciences, University of Saskatchewan, Saskatoon, Saskatchewan, Canada.

^7^ Department of Biology & Chemistry and State Key Laboratory in Marine Pollution, City University of Hong Kong, Kowloon, Hong Kong, SAR, China

^8^ School of Biological Sciences, University of Hong Kong, Hong Kong, SAR, China

^9^ State Key Laboratory of Pollution Control and Resource Reuse, School of the Environment, Nanjing University, Nanjing, People’s Republic of China

^10^ Department of Biology, Hong Kong Baptist University, Hong Kong, SAR, China

*Corresponding author: Jingchun Tang

Tel.: +86-22-83614117, Fax: +86-22-83614117

E-mail address: tangjch@nankai.edu.cn

**Table S1.** Proportions of THCs contributed by SHs and AHs.

| Samples | | SHs Proportions | AHs Proportions |
| --- | --- | --- | --- |
| Oil -producing  zone | S1 | 28.8% | 13.9% |
|  | S2 | 19.4% | 14.7% |
|  | S3 | 15.7% | 15.1% |
|  | S4 | 13.2% | 13.3% |
|  | S5 | 37.3% | 8.1% |
|  | Mean | 23.3% | 13.3% |
|  | | | |
| Residential  zone | S6 | 7.6% | 17.3% |
|  | S7 | 26.2% | 8.9% |
|  | S8 | 15.4% | 3.1% |
|  | S9 | 32.7% | 10.0% |
|  | Mean | 20.5% | 8.12% |
|  | | | |
| Oil-refinery  and  transportation  zone | S10 | 38.7% | 9.3% |
|  | S11 | 27.1% | 24.3% |
|  | S12 | 33.6% | 7.8% |
|  | S13 | 38.1% | 15.3% |
|  | S14 | 32.3% | 9.2% |
|  | Mean | 34.1% | 13.2% |

**Table S2.** Relative abundances of *AlkB* and *Nah* in 14 soils collected in Dagang Oilfield. Abundances of genes were normalized to the total 16S rDNA.

| Samples | | Relative abundance (Copy of oil-degrading genes/Copy of 16S rDNA) | |
| --- | --- | --- | --- |
|  |  | *AlkB* | *Nah* |
| Oil-producing  zone | S1 | (4.8±0.3)×10^-3^ | (1.7±0.2)×10^-3^ |
|  | S2 | (2.9±0.2)×10^-3^ | (3.8±0.4)×10^-2^ |
|  | S3 | (3.2±0.4)×10^-3^ | (1.5±0.2)×10^-2^ |
|  | S4 | (3.1±0.3)×10^-3^ | (2.6±0.1)×10^-2^ |
|  | S5 | (1.1±0.1)×10^-3^ | (4.4±0.3)×10^-3^ |
|  | Mean | (3.0±0.4)×10^-3^ | (1.7±0.1)×10^-2^ |
| Residential  zone | S6 | (5.3±0.5)×10^-4^ | (3.3±0.2)×10^-3^ |
|  | S7 | (6.3±0.4)×10^-4^ | (2.8±0.3)×10^-3^ |
|  | S8 | (4.4±0.2)×10^-4^ | (1.0±0.1)×10^-3^ |
|  | S9 | (1.5±0.3)×10^-5^ | (2.9±0.4)×10^-4^ |
|  | Mean | (4.0±0.3)×10^-4^ | (1.8±0.2)×10^-3^ |
| Oil-refinery  and  transportation  zone | S10 | (2.3±0.2)×10^-4^ | (9.4±0.5)×10^-3^ |
|  | S11 | (1.7±0.3)×10^-3^ | (1.8±0.2)×10^-3^ |
|  | S12 | (3.8±0.5)×10^-3^ | (5.3±0.2)×10^-2^ |
|  | S13 | (1.4±0.2)×10^-3^ | (3.9±0.4)×10^-3^ |
|  | S14 | (4.2±0.1)×10^-3^ | (8.1±0.5)×10^-2^ |
|  | Mean | (2.3±0.3)×10^-3^ | (3.0±0.1)×10^-2^ |

**Table S3.** Diversity indexes of the microbial communities in soils of the Dagang Oilfeild, China, based on DGGE analysis

| Soil sampling sites | Richness | Shannon-Wiener Index | Uniformity Index |
| --- | --- | --- | --- |
| 1 | 10 | 2.437 | 0.95 |
| 2 | 9 | 2.478 | 0.938 |
| 3 | 12 | 2.77 | 0.977 |
| 4 | 6 | 2.382 | 0.928 |
| 5 | 11 | 2.789 | 0.947 |
| 6 | 3 | 1.538 | 0.955 |
| 7 | 5 | 1.501 | 0.932 |
| 8 | 14 | 2.126 | 0.983 |
| 9 | 10 | 2.533 | 0.959 |
| 10 | 11 | 2.831 | 0.979 |
| 11 | 9 | 1.987 | 0.904 |
| 12 | 11 | 2.515 | 0.907 |
| 13 | 5 | 2.138 | 0.891 |
| 14 | 6 | 1.755 | 0.901 |

**Table S4.** The fragment length of PCR primers and annealing temperature conditions

| Primer | Proteins targeted | Sequence(5’-3’) | Amplicon size (bp) | PCR annealing  temperature(°C) | Reference |
| --- | --- | --- | --- | --- | --- |
|  |  |  |  |  |  |
| *AlkB* | Alkane monooxygenases | AACTACMTCGARCAYTACGG | 100 | 50 | 44 |
|  |  | TGAMGATGTGGTYRCTGTTCC |  |  |  |
|  |  |  |  |  |  |
| *Nah* | Naphthalene dioxygenase | ACTTGGTTCCGGAGTTGATG | 136 | 57 | 45 |
|  |  | CAGGTCAGCATGCTGTTGTT |  |  |  |
|  |  |  |  |  |  |
| 16S rDNA | - | CGGTGAATACGTTCYCGG | 126 | 58 | 46 |
|  |  | GGWTACCTTGTTACGACTT |  |  |  |

**Figure S1.** Standard curves of *AlkB* gene (a), *Nah* gene (b) and 16S rDNA. Standard curve of *AlkB* gene was created as Ct=-3.017 lgC+38.822, and the coefficient of determination (R^2^) was 0.996 with an [amplification](app:ds:amplification) efficiency (E) of 114.5%. Standard curves for the *Nah* degradation gene was determined as Ct=-2.705 lgC+35.675 (R^2^=1, E=134.2%). Standard curve for 16S rDNA was Ct=-3.279 lgC+41.622 (R^2^=0.999, E=101.8%).
